# Elongated Flexuous Plant Virus-Derived Nanoparticles Functionalized for Autoantibody Detection

**DOI:** 10.3390/nano9101438

**Published:** 2019-10-10

**Authors:** Carmen Yuste-Calvo, Mercedes López-Santalla, Lucía Zurita, César F. Cruz-Fernández, Flora Sánchez, Marina I. Garín, Fernando Ponz

**Affiliations:** 1 Centro de Biotecnología y Genómica de Plantas, Universidad Politécnica de Madrid-Instituto Nacional de Investigación y Tecnología Agraria y Alimentaria (CBGP, UPM-INIA), Campus Montegancedo, Autopista M-40, km 38. Pozuelo de Alarcón, 28223 Madrid, Spain; carmen.yus.cal@gmail.com (C.Y.-C.); lucia.zurita@inia.es (L.Z.); cesarfcruzf@gmail.com (C.F.C.-F.); florasanchez@telefonica.net (F.S.); 2 Division of Hematopoietic Innovative Therapies, Centro de Investigaciones Energéticas, Medioambientales y Tecnológicas (CIEMAT) and Centro de Investigación Biomédica en Red de Enfermedades Raras (CIBER-ER), 28040 Madrid, Spain; Mercedes.LopezSantalla@externos.ciemat.es (M.L.-S.); marina.garin@ciemat.es (M.I.G.); 3 Advanced Therapy Unit, Instituto de Investigación Sanitaria Fundación Jiménez Díaz (IIS-FJD/UAM), 28040 Madrid, Spain

**Keywords:** VNPs, Hsp60, IBD, autoantibody, inflammation, diagnosis

## Abstract

Nanoparticles derived from the elongated flexuous capsids of *Turnip mosaic virus* (TuMV) have been shown to be efficient tools for antibody sensing with a very high sensitivity if adequately functionalized with the corresponding epitopes. Taking advantage of this possibility, TuMV virus-like particles (VLPs) have been genetically derivatized with a peptide from the chaperonin Hsp60, a protein described to be involved in inflammation processes and autoimmune diseases. Antibodies against the peptide have been previously shown to have a diagnostic value in at least one autoimmune disease, multiple sclerosis. The functionalized Hsp60-VLPs showed their significant increase in sensing potency when compared to monoclonal antibody detection of the peptide in a conventional immunoassay. Additionally, the developed Hsp60-VLPs allowed the detection of autoantibodies against the Hsp60 peptide in an in vivo mouse model of dextran sodium sulfate (DSS)-induced colitis. The detection of minute amounts of the autoantibodies allowed us to perform the analysis of their evolution during the progression of the disease. The anti-Hsp60 autoantibody levels in the sera of the inflamed mice went down during the induction phase of the disease. Increased levels of the anti-HSP60 autoantibodies were detected during the resolution phase of the disease. An extension of a previously proposed model for the involvement of Hsp60 in inflammatory processes is considered, incorporating a role for Hsp60 autoantibodies. This, and related models, can now be experimentally tested thanks to the autoantibody detection hypersensitivity provided by the functionalized VLPs.

## 1. Introduction

The use of viral nanoparticles (VNPs) for biomedical applications has become a new tool in theranostics. Specifically, VNPs derived from plant viruses offer advantages in terms of biosafety since they are only plant pathogens and also because of plant virus variability in nature, size, and structure, that allows a specific design of VNPs depending on the application [1,2,3,4,5,6,7,8,9,10,11,12,13]. We work with *Turnip mosaic virus* (TuMV), a virion with an elongated and flexuous structure, 700 nm long and 12 nm wide, with *ca*. 2000 copies of the coat protein in each particle, from which multifunctional VNPs can be obtained by genetic fusion and/or chemical conjugation to the coat protein (CP) [14,15,16,17]. One potential application is their use as tools for the detection of circulating antibody levels that frequently are too low to be detected by conventional detection methodologies, thus contributing to improve the diagnosis, progression, and/or prognosis of immunity-mediated pathologies, especially at the early stages of the disease when no clear symptoms of the inflammation are evident.

Inflammatory bowel disease (IBD) is a pathology encompassing two chronic inflammatory disorders; Crohn′s disease, characterized by transmural inflammation usually affecting the terminal ileus and/or the large intestine; and ulcerative colitis, where inflammation occurs in the lining of the colon mucosa [18,19]. In these pathologies, our research focuses on diagnosis improvement and the development of personalized therapy, in order to achieve improvement in the patients’ quality of life. On the other hand, we seek to gain new knowledge about the multifactorial pathogenesis of these disorders, for which there are several representative animal models, capable of mimicking the symptomatology and pathology of the disease. For IBD, a murine model induced by dextran sodium sulfate (DSS) has been developed [20,21]. This compound, when administered in drinking water, induces inflammation of the colon mucosa, as well as ulceration, which leads to severe weight loss and, in extreme cases, lethality.

We have explored the deployment of Heat Shock Protein 60 (Hsp60) as a possible autoantigen in IBD, due to the implication of Hsp60 in inflammatory processes [22,23,24,25,26,27,28,29,30,31], including IBD [32,33,34,35]. Heat shock proteins are a broad family of molecular chaperons capable of reacting to cellular stress, especially in thermal changes. However, they also respond in other stress situations, such as tissue damage, cellular injury, or heavy metal poisoning. Hsp60, despite being a protein whose main function occurs in the mitochondria, is able to act in the cytoplasm modulating the immune response associated with inflammation. Both the complete protein and the peptides derived therefrom can act as natural regulators of the inflammatory reaction together with other cytokines, chemokines, and autoantibodies, regulating the immune system [30,32,36,37,38,39]. The activity of Hsp60 has been associated with various autoimmune and inflammatory pathologies, such as atherosclerosis, diabetes, rheumatoid arthritis, or multiple sclerosis [23,25,29,40,41,42].

Alterations in the levels of anti-Hsp60 autoantibodies have been described in several inflammatory pathologies [22,23,26,27,37], making the autoantibody detection a novel strategy with potential application for the diagnosis, progression, and/or prognosis of the disease. In these studies, exhaustive analyses by peptide arrays have allowed the identification of certain epitopes for the design of specific VNPs where the election of a peptide with diagnostic value is the key to success. Based on these premises, we have designed functionalized TuMV VNPs with an epitope, described previously in multiple sclerosis [40]. The multimeric presentation of this epitope on TuMV VNPs (Hsp60-virus-like particles (VLPs)) allowed us the quantitative detection of anti-Hsp60 autoantibodies in an in vivo model of intestinal inflammation induced by DSS. The high detection levels of the developed Hsp60-VLPs may represent a novel tool that can be used for the diagnosis, progression, and/or prognosis in inflammation-mediated disorders.

## 2. Materials and Methods

### 2.1. Construction of CP and Hsp60-CP Expression Plasmids

Constructs corresponding to infectious clones were obtained by designing synthetic genes (GeneArt), based on the p35Tunos-Vec01-Nat1 sequence, as shown in Figure S1, derived from an original TuMV infectious clone [43]. Synthetic DNA consisted of a partial NIb gene, starting with the sequence corresponding to the restriction site *Mlu* I, followed by alanine codon (first CP amino acid) to maintain the protease recognition sequence, followed by the sequence encoding the Hsp60 peptide, which comprises amino acids 301–320 of human Hsp60 (KAPGFGDNRKNQLKDMAIAT, sequence conserved in mice), and the restriction site *Nae* I, corresponding to the first two CP amino acids. The DNA was digested with *Mlu* I and *Nae* I. The resulting fragment was purified. Vector p35Tunos-vec01-Nat1 was also digested with these two restriction enzymes, and the two fragments ligated together to obtain the recombinant vector with the sequence encoding Hsp60 peptide.

For VLP expression plasmids, PCR (GeneAmp® PCR System 9700, Applied Biosystems, CA, United States) was performed, amplifying the whole modified CP with two extra codons—one corresponding to the initial methionine, and a STOP codon. The nucleotide sequence CACC was also added at the 5’ end to allow directional cloning into a pENTR-D-TOPO vector, as shown in Figure S2. Then the fragment was cloned into a pEAQ-HT vector, as shown in Figure S3, by Gateway cloning with LR clonase enzyme.

### 2.2. Production and Purification of VLPs

For VLP production, the pEAQ construct was transformed into *Agrobacterium tumefaciens* LBA4404 for agroinfiltration mediated transitory expression in *Nicotiana benthamiana* plants [44,45]. The same procedure was followed for non-modified VLPs. Plant growth, *Agrobacterium* culture preparation, agroinfiltration, tissue harvesting, and VLP purification were performed as previously described by us [16,17].

### 2.3. VLP Characterization

Characterization of assembled purified VLPs was performed by SDS-PAGE, western blot and transmission electron microscopy (TEM, ICTS-CNME, Madrid, Spain). 

The conditions for SDS-PAGE and western blot were as described [16]. Anti-Hsp60 D307 antibody was from Invitrogen.

To check structural integrity, TEM was performed. Electron microscopy grids (400 mesh copper, carbon coated) were coated at room temperature for 15 min with a 10 µL drop of VLPs diluted at 0.02 mg/mL final concentration (50 mM borate buffer, pH 8.1), and washed with buffer. Finally, the grids were rinsed with distilled water and stained with 2% uranyl acetate for 2 min. Samples were examined on a transmission electron microscope (JEM JEOL 1010, Tokyo, Japan).

### 2.4. IBD Murine Model

Adult C57BL/6J (8 weeks-old) mice used in these studies were obtained from the Jackson Laboratory (ref. 000664). The regulations concerning experimental animal welfare (RD 223/1998 and Directive 2010/63/EU protocols) were followed. The ethics committee for animal research of the CIEMAT (Proex. 414/15) and Comunidad de Madrid (based on the RD 53/2013) reviewed and approved all protocols.

The IBD was induced in mice by dextran sodium sulfate (DSS, MP Biochemicals), a sulphated polymer cytotoxic on intestinal epithelial cells and macrophages. In addition, enteral DSS favors Gram-negative anaerobic bacteria increases, which together with the erosive potential on the intestinal barrier and the macrophages’ inappropriate response, would lead to the appearance of intestinal lesions [20,21]. This model reproduces the clinical, histopathological, and immune characteristics observed in humans, inducing chronic colitis associated with diarrhea and weight loss.

The chemical compound was administered in drinking water at 1.35% (*w*/*v*) over 7 days *ad libitum* and disease progress was determined by monitoring mouse weight and the number of granulocytes in peripheral blood by an automated blood cell-counter (Abacus, Diatron, Budapest, Hungary). Normal distribution was analyzed by the Shapiro–Wilks test.

Non-parametric techniques (Mann–Whitney U test) were used. Autoantibody levels were determined in mouse sera, obtained at different times after disease induction. All trials were of a “simple blind” type.

### 2.5. Immunoassays

Indirect enzyme-linked immunosorbent assays (ELISA) were performed in order to evaluate the sensitivity provided by the VLPs, in comparison with the whole Hsp60 protein and free peptide at equal peptide amounts, and also to measure autoantibody levels in peripheral blood. Plates (Nunc MaxiSorp) were coated with 1 μg purified VLPwt or Hsp60-VLPs, resuspended in 50 mM sodium carbonate buffer, pH 9.6; or equivalent amounts of Hsp60 protein or peptide, resuspended in the same buffer and incubated overnight at 4 °C. Plates were washed intensively, and incubated for 1 h with commercial antibodies [Hsp60 (D307) Antibody 4870S, CellSignal and Anti-Hsp60 antibody (ab46798), Abcam; different dilutions in phosphate-buffered saline, pH 7.4, 0.05% Tween 20 (*v*/*v*), 2% PVP-40 (*w*/*v*)] or overnight at 4 °C for mice sera (diluted 1:100 in the same buffer). Then, secondary antibodies, diluted 1:1500 in the same buffer, were added and incubated for 1 h at room temperature. Color was developed by alkaline phosphatase reaction and detected after addition of *p*-nitrophenylphosphate. Absorbance was measured at 405 nm (TECAN Genios Pro). Differences between both coating-VLPs were calculated, since this would be the measure attributable to autoantibodies against Hsp60. The normalization process was done as follows. A serum pool was made using the sera from healthy mice and this pool was included in all the plates in order to calculate the background of each plate. The normalized values of autoantibodies directed against Hsp60 in the DSS serum samples were obtained by taking into account the mean value of the healthy serum pool in each plate and the mean value of the healthy serum pool from all the plates, in order to minimize the effects of the variation in the background signal. The data obtained were analyzed based on the non-parametric Mann–Whitney U test.

## 3. Results

### 3.1. Production and Characterization of Hsp60-VLPs

Our first Hsp60-VNP derivatization approach was the production of virions genetically modified with the Hsp60-peptide fused to the CP N-terminus. However, the insertion of the peptide interfered with the infectivity of the modified virus, so this strategy was discarded. Starting out from the virion construct, a second construction strategy was followed to generate now modified VLPs. 

**Figure 1:**
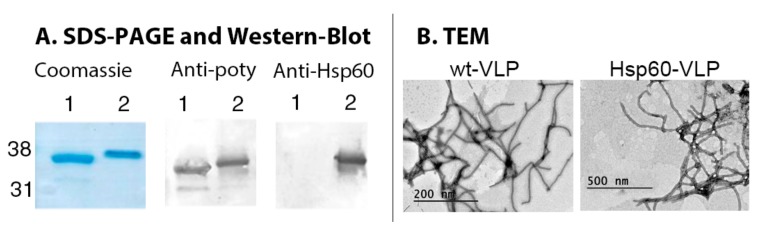
Hsp60-virus-like particles (VLPs) characterization. (**A**) SDS-PAGE and western blot of wild type VLPs (1) and Hsp60-VLPs (2). (**B**) Micrographs of wt-VLPs and Hsp60-VLPs, where particles of about 700 nm are shown.

VLPs modified by genetic fusion of the Hsp60-peptide to the CP were made by transient expression of the fusion protein CP-Hsp60 peptide in plants. As shown before [16], the fusion protein will self-assemble into VLPs within plant cells. Once purified nanoparticles were obtained, they were characterized by SDS-PAGE, western-blot assays, and transmission electron microscopy, as shown in Figure 1. 

The results obtained showed a modified electrophoretic mobility for Hsp60-VLPs CP, consistent with a molecular weight increase of approximately 2.5 kDa. This fusion protein was detected specifically not only by antibodies directed against the virus, but also by those directed against the Hsp60 protein. Finally, TEM structural characterization showed elongated and flexuous particles with similar length and thickness than non-modified particles, so no detectable significant changes in VLP structure were found after peptide insertion.

### 3.2. Increased Sensitivity of Hsp60 Peptide Detection by Specific Hsp60 Antibodies

**Figure 2:**
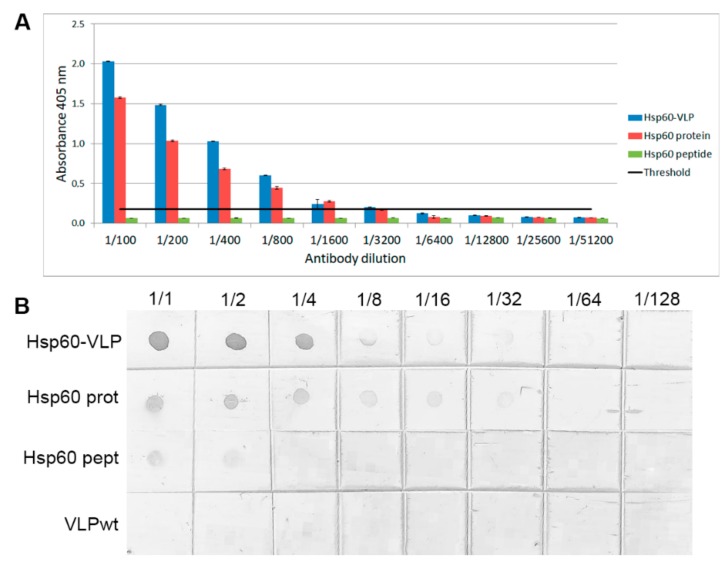
Specificity and sensitivity assays. (**A**) ELISA using monoclonal Hsp60 antibody dilutions, comparing Hsp60-VLPs with free Hsp60 peptide and Hsp60 protein. The black line indicates the positive signal threshold and the standard error is also shown (three replicates). (**B**) Dot blot using the same components, and also VLPs-wt.

Multimeric peptide presentation on the surface of TuMV VNPs increases its detectability by specific antibodies in conventional immunoassays like ELISA [15,17,46]. To evaluate this in the Hsp60-VLPs construct, ELISA and dot-blot assays were performed using three types of presentations: Hsp60-VLPs, free Hsp60 peptide, and complete Hsp60 protein, as shown in Figure 2. Assays were performed using equal peptide amounts presented in the three forms. Two different antibodies were used to assess the response, a monoclonal antibody that specifically recognizes an epitope within the peptide 301–320, and a polyclonal antibody against complete Hsp60.

Using the monoclonal antibody, it was found that free Hsp60 peptide was hardly recognized, while both the complete Hsp60 protein and the Hsp60-VLPs were easily detected, with Hsp60-VLPs being the most sensitive system.

Hsp60-VLP detectability, compared with the complete protein using polyclonal antibodies, was evaluated, as shown in Figure S4. In this case, complete Hsp60 protein presents greater sensitivity, possibly due to the higher number of epitopes present on its complete surface.

The results obtained show that, although the complete HSP60 protein contains various epitopes, the multimeric presentation in VNPs of a defined epitope allows differential detection by measurement of specific autoantibodies in biological fluids, such as peripheral blood serum. These results highlight the Hsp60-VLPs potential for specific antibody detection, with the epitope election being the key to develop TuMV-VNPs as a diagnostic tool.

### 3.3. Hsp60-VLPs Autoantibody Detection

**Figure 3:**
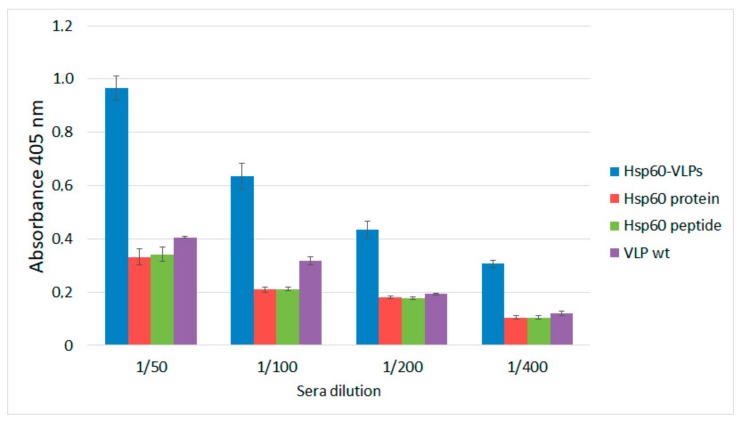
Anti-HSP60 autoantibody detection in the sera of healthy mice by ELISA, using different antigen-presentation platforms; Hsp60-VLP (blue), complete protein Hsp60 (red), Hsp60 peptide (green), and VLPwt (purple) and at different sera dilutions. Standard error bars shown (three replicates).

Once sensitivity and specificity were shown through different immunoassays, the potential of Hsp60-VLPs as a diagnostic tool of autoimmune pathologies, exemplified by IBD, was tested, measuring autoantibodies against Hsp60 in a pool of control sera, as shown in Figure 3. In these pathologies, autoantibody levels are usually very low at early stages, but early diagnosis by detecting these low levels would improve patients’ prognosis.

To compare the data obtained with all detection systems (Hsp60-VLPs, complete Hsp60 protein, and free peptide), an ELISA was developed with a pool of sera from healthy, non-inflamed mice, in order to determine physiological levels of anti-Hsp60 autoantibodies. Unmodified VLPs (VLPwt, not displaying the Hsp60 peptide) were used as a negative control. 

The results showed that only with the Hsp60-VLPs could the anti-Hsp60 autoantibodies be detected above the background of the plate and negative controls in healthy mice sera. Therefore, the high ability of Hsp60-VLPs to detect autoantibody levels, undetectable by conventional procedures such as ELISA, was demonstrated. This result indicates that VLPs can be used as a tool to detect variations in the levels of anti-Hsp60 autoantibodies in mice sera when the traditional methodologies based on the complete Hsp60 protein cannot be detected, even at basal autoantibody levels in non-inflamed mice.

### 3.4. Hsp60-VLPs for Autoantibody Detection in DSS-Induced Colitis

Due to the proven ability of Hsp60-VLPs to detect low levels of autoantibodies in the sera samples, potential diagnostic applications were assessed. Since Hsp60 is deeply involved in inflammatory processes [23,25,47], a murine model of the inflammatory bowel disease (IBD) induced by DSS was selected for further studies [20,21]. DSS-induced colitis is a favorite model for chemically induced colitis because it is a simple one and highly similar to human IBD. DSS induces intestinal inflammation through a yet non-deciphered mechanism, although it probably results from damage to the epithelial monolayer lining the large intestine. This lesion would allow dissemination of proinflammatory intestinal contents (e.g., bacteria and their products) into the tissue underneath [20].

**Figure 4:**
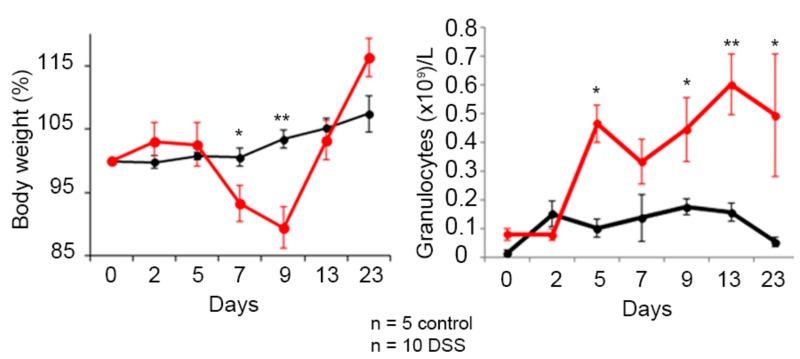
Colitis status of mice treated with dextran sodium sulfate (DSS) in the drinking water. Assessment of the pathology progression by determination of body weight loss and granulocyte levels in peripheral blood. Healthy mice are shown in black and the group of mice treated with DSS is shown in red. The mean and the standard error of the mean are shown, as well as those significant results according to the nonparametric Mann–Whitney U test (* *p* ≤ 0.05 and ** *p* ≤ 0.01).

The evolution of Hsp60 autoantibody levels in the inflammatory model induced by DSS was studied. The progress of the pathology, as well as the recovery after removing the chemical agent, was determined by monitoring the body weight and granulocytes levels in peripheral blood, as shown in Figure 4.

The results show a significant decrease in body weight of mice treated with DSS, significant at day 7 and 9. This decrease continues until the DSS was withdrawn, after which the mice recovered weight and reverted to a non-pathological state. Regarding granulocyte levels, there was an increase in blood, in this case significantly at 5 days. In this case the reversal begins later, several days after withdrawing DSS.

**Figure 5:**
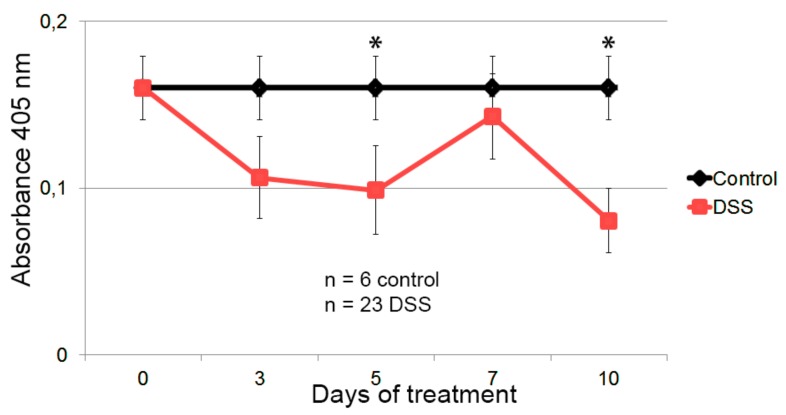
Detection of anti-HSP60 autoantibody in the sera of the healthy and DSS-colitic mice. Healthy mice are represented in black, and in red are mice treated with DSS in the drinking water for 7 days. The levels of anti-HSP60 autoantibodies in the control group has been defined as the average value of all the mice in the control group, including the values of the DSS-treated colitic mice at time 0. Mean and standard error of the mean are shown, as well as those significant results according to the Mann–Whitney U nonparametric test (* *p* ≤ 0.05).

To evaluate the potential of Hsp60-VLPs as a novel tool in the knowledge of the inflammatory pathology, an ELISA was performed. Autoantibody levels measured with the complete Hsp60 protein were not detectable, as shown in Figure S5, so only data obtained with Hsp60-VLPs and VLPs-wt were used to assess autoantibody levels in sera of the mice diluted 100-fold. Data were normalized, eliminating the signal attributable to the background which could be originated by the viral component of nanoparticles, and the results were analyzed, as shown in Figure 5.

The results showed a marked decrease in anti-Hsp60 autoantibody levels following the progress of inflammation at 5 and 10 days (significant according to the Mann–Whitney U nonparametric test), maintaining higher levels in healthy individuals and the basal levels at day 0 in the DSS group. These results demonstrate TuMV functionalized VNPs as a hypersensitive tool in the progression analysis of the inflammation, being able to detect levels of autoantibodies in cases where conventional methodology is not capable.

## 4. Discussion

In the diagnosis of autoimmune pathologies, current techniques face two main problems, especially when diagnosis is made by detecting specific autoantibodies in serum—background and low autoantibody levels. The high background is due to numerous substances present in serum, and low autoantibody levels occur at early stages of pathology development, a time in which a proper diagnosis would allow early treatment, and a better patient prognosis [48,49]. This is in addition to problems associated with the use of biological samples, such as peripheral blood sera. There is also an added problem when specific epitopes are needed as the basis of a detection system. These epitopes, small peptides, are unable to adhere efficiently to test plates [50], thus leading to very low sensitivity compared to systems deploying high molecular weight proteins in which the handicap is the presence of numerous epitopes, not all of them with diagnostic value. All these inconveniences in conventional methodologies make it increasingly convenient to develop new approaches for a highly sensitive technique, capable of detecting very low levels of autoantibody, or rather small changes, in diluted sera.

To address these challenges, the use of nanoparticles as a multimeric presentation system is an alternative approach, which should provide high sensitivity and solve the epitope–adhesion problem [6,9,51,52]. We have previously shown that functionalized nanoparticles derived from TuMV significantly increased antibody detection with respect to conventional systems [16,17,46]. Although this system has not been used in the diagnosis, progression, and/or prognosis of specific pathologies yet, its potential makes it a good candidate for the design of a hypersensitive analysis system by the detection of serum autoantibodies.

To test TuMV VNPs as a new tool for autoantibody detection, Hsp60 was chosen as an antigen. This protein is involved in numerous inflammatory mechanisms, also related to autoimmune pathologies [27,30,32,33,35,38,40]. Regarding Hsp60 specific commercial antibodies, sensitivity tests were made by comparing the Hsp60-VLPs’ detection capacity with respect to the complete recombinant Hsp60 protein. The results showed that, despite not showing a greater sensitivity in polyclonal antibody detection as shown in Figure S1, VLPs sensed epitope-specific monoclonal antibodies with greater sensitivity, as shown in Figure 2. This may be due not only to multimeric presentation in the nanoparticle, but also to a better epitope-exposure on the VLP surface with respect to the complete Hsp60 protein.

According to these results, and because this peptide was chosen due to its diagnostic potential in autoimmune pathologies [49], an evaluation of its efficacy in IBD diagnosis in a DSS-induced colitis murine model [20,21] was decided. In the sera of these mice, nanoparticles exhibited greater detection power not only compared to the free peptide, but also to the complete Hsp60, which was not able to detect autoantibodies under the same conditions. As mentioned above, the enhanced sensitivity of the Hsp60-VLPs could be provided not only because of a multimeric presentation of the chosen epitope, but also because of its exposure on the particle surface since the epitope may have a more internal location in the complete Hsp60 but be exposed on the surface in VLPs. This approach should allow the development of sets of designed nanoparticles for each disease, selecting peptides with diagnostic value present in various autoantigens, and presenting them on the same multifunctionalized nanoparticle [15], improving diagnosis, progression, and prognosis of autoimmune diseases. 

**Figure 6:**
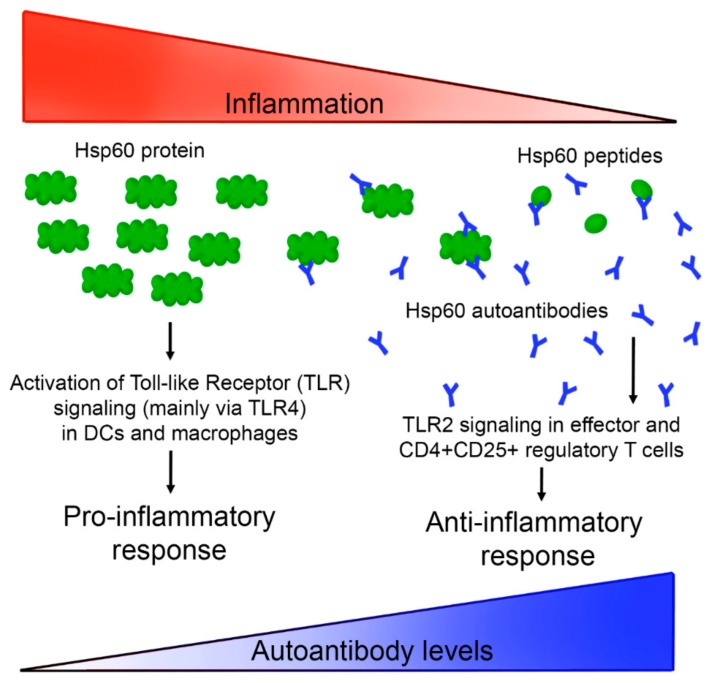
A possible Hsp60 autoantibody regulation system based on a previously described model [30]. High Hsp60 protein levels, related with endogenous proteins and/or similar chaperonins present in microbiota or other pathogens, would activate inflammation pathways, which in turn would induce the production of autoantibodies against Hsp60, neutralizing the antigen, and generating different peptides that activate an anti-inflammatory response.

Thanks to the high sensitivity shown by Hsp60-VLPs, it was possible to evaluate the evolution of autoantibody levels with disease progress in a murine model. In this case, and against expectations, autoantibody levels decreased concomitantly with the inflammatory process, as shown in Figure 5, suggesting a new view regarding the mechanism of action of Hsp60 (and its autoantibodies) in the inflammatory process. According to these results, anti-Hsp60 autoantibodies could be considered as biomarkers of a non-pathological inflammation state, participating in immune homeostasis as “immunomodulators”, recently called “immunculus” (“immune homunculus”), as it was already described for other molecules and autoantibodies [36,39,53]. According to this model, and taking into account studies relating a decrease in autoantibody levels with autoimmune disease development [25,26,32,41,54,55], it seems that autoantibodies in healthy individuals may play a role as a mechanism to regulate inflammation, avoiding a pathological inflammatory response caused by Hsp60. This protein, and peptides derived thereof, have been implicated in pro- and anti-inflammatory processes, and a model has been previously proposed [30]. The results presented in this study allow us to extend tentatively this model, involving now a role for Hsp60 autoantibodies in the progression of the inflammation. A general view of such an extended model is presented in Figure 6. The presence of large amounts of Hsp60 protein (and/or other similar chaperonins present in intestinal microbiota) would be related to an activation of the inflammatory response, triggering the production of autoantibodies. These autoantibodies would act as neutralizers, regulating protein levels, and thereby deactivating the inflammatory response and even activating new anti-inflammatory pathways. The regulatory balance of these pathways in both directions would be essential to maintain a non-pathological physiological state. This proposed process would imply that, when an inflammatory process is induced by external agents, as occurs in experimental colitis induced by DSS, inflammation would be accompanied by a decrease in the levels of regulatory autoantibodies.

All the ideas raised would place Hsp60 as a study target. In this case, TuMV VLPs allowed the identification of possible markers in inflammatory pathologies, being able to generate diagnostic systems of high sensitivity and specificity, useful to identify new diagnostic and therapeutic targets. In addition, considering cases such as Hsp60, where autoantibodies can act as regulatory agents, functionalized VLPs could be used not only as a diagnostic tool, but also in therapy for immunization, where the great immunogenic power of VNPs from TuMV has been demonstrated [17]. This would be related to other studies connecting autoantibodies and inflammation, in which immunization with the protein in patients induced symptomatic amelioration [25,41,56]. This work demonstrates that VNPs are a good alternative in the biomedical field, acting as a functionalizable platform for different applications, from diagnosis to treatment. In this context, exploring the possibility of using several epitopes from Hsp60 (or even from other new autoantigens) in multifunctional nanoparticles, would open the door to new theranostic tools to improve prognosis of patients with autoimmune pathologies.

## 5. Conclusions

This study lays the groundwork for the development of new theranostic tools, based on viral nanoparticles functionalized with specific epitopes involved in autoimmune pathologies, allowing the creation of new systems for autoantibody-based specialized diagnosis and for targeted treatments.

## Supplementary Materials

The following are available online at https://www.mdpi.com/2079-4991/9/10/1438/s1, Figure S1. Vector p35Tunos-Vec01-NAT1. Figure S2. Vector pENTR/D-TOPO. Figure S3. Vector pEAQ-HT-Dest1. Figure S4. Sensitivity comparison between different detection platforms using commercial antibodies. Figure S5. Autoantibody levels in sera measured with Hsp60-VLPs (blue) and Hsp60 protein (red).
