# Supplementary material for: Elongated Flexuous Plant Virus-Derived Nanoparticles Functionalized for Autoantibody Detection
Source: Nanomaterials (Basel). 2019 Oct 10;9(10):1438. doi: 10.3390/nano9101438 (PMC6835482; doi:10.3390/nano9101438)
Supplement: Supplementary file 1 [file nanomaterials-09-01438-s001.pdf]

Supplementary material

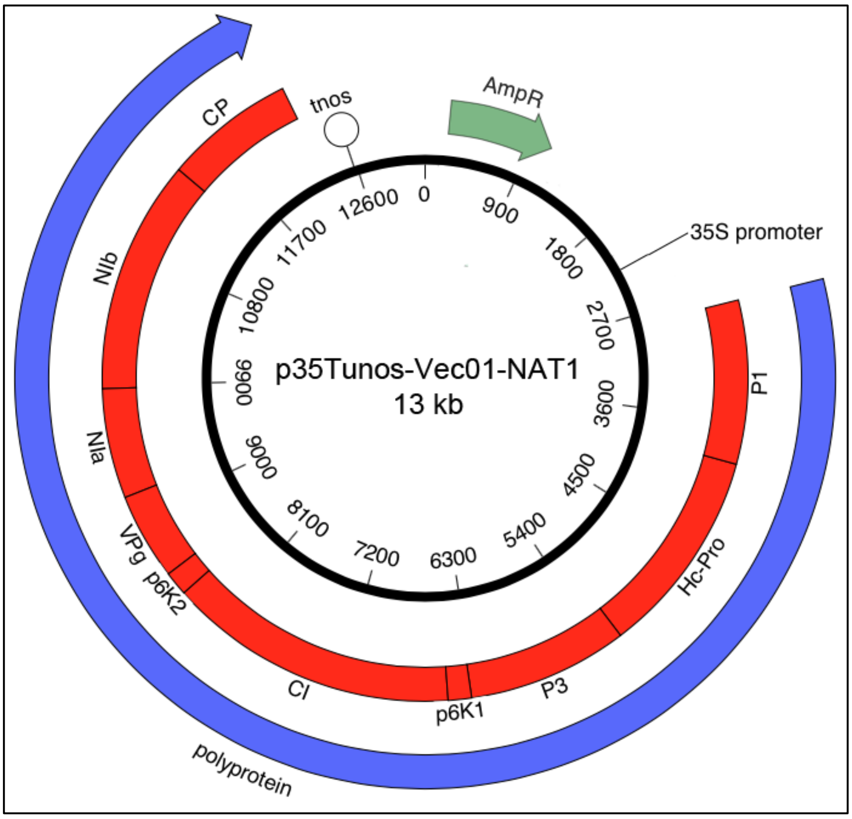

Figure S 1. Vector p35Tunos-Vec01-NAT1. Both the *35S* promoter and the *nos* terminator are shown, in addition to the ORF encoding the virus polyprotein and the ampicillin resistance gene.

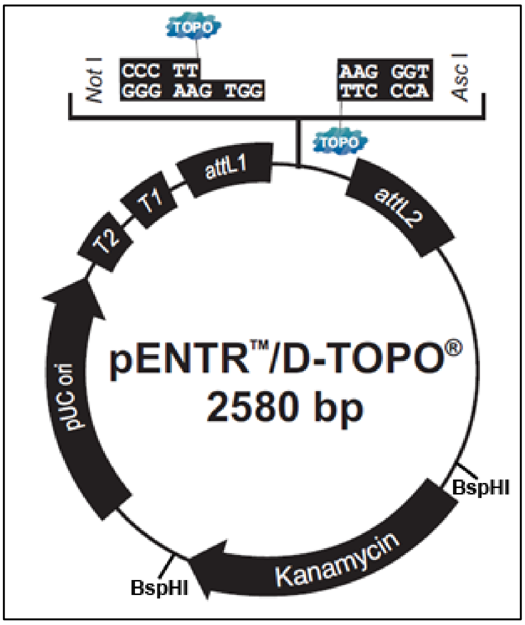

Figure S 2. PENTR™/D-TOPO® vector. The GTGG sequence is for directional cloning, where the synthetic gene is inserted, flanked by the restriction sites *Not* I and *Asc* I. Also shown are sequences corresponding to terminators *T1* and *T2*, the origin of replication *pUC*, the sequences for Gateway cloning *attL1* and *attL2* and Kanamycin resistance gene flanked by restriction site *Bsp* HI.

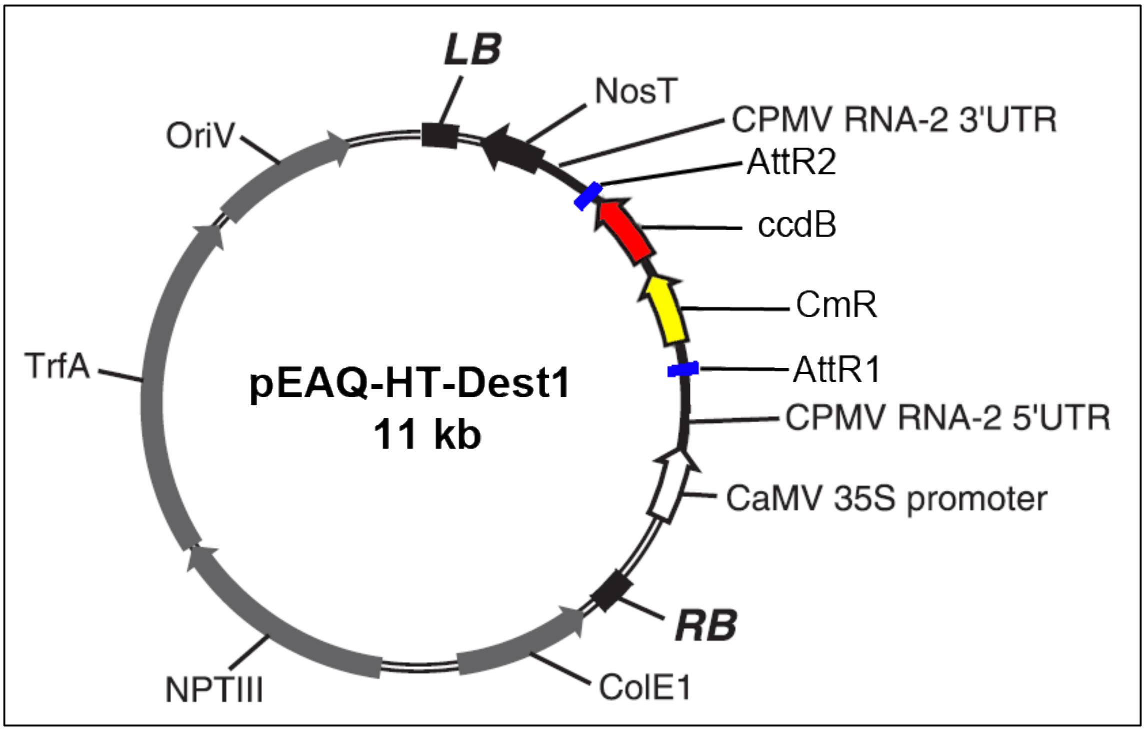

Figure S 3. Vector pEAQ-HT-Dest1. The region involved in Gateway recombination is comprise between *UTR* regions. The chloramphenicol resistance gene (*CmR*) is shown in yellow, the cell proliferation control gene (*cddB*) in red, both flanked by *attR* regions (blue), which are recombined with the *attL* regions of pENTR/D-TOPO vector for Gateway cloning.

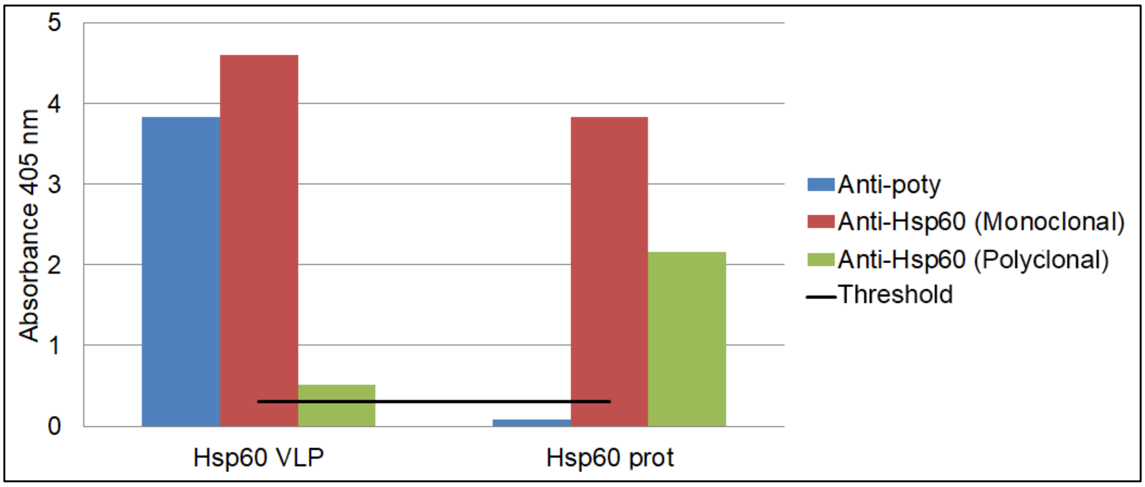

Figure S 4. Sensitivity comparison between different detection platforms using commercial antibodies. In blue, antibody against potyviruses, detecting the VLPs. In red, monoclonal antibody directed against the region close to amino acid D307, included in the peptide presented in the functionalized Hsp60-VLPs. In green, polyclonal antibody for Hsp60 protein. Black line is the detection threshold. Same results were obtained in three experiments.

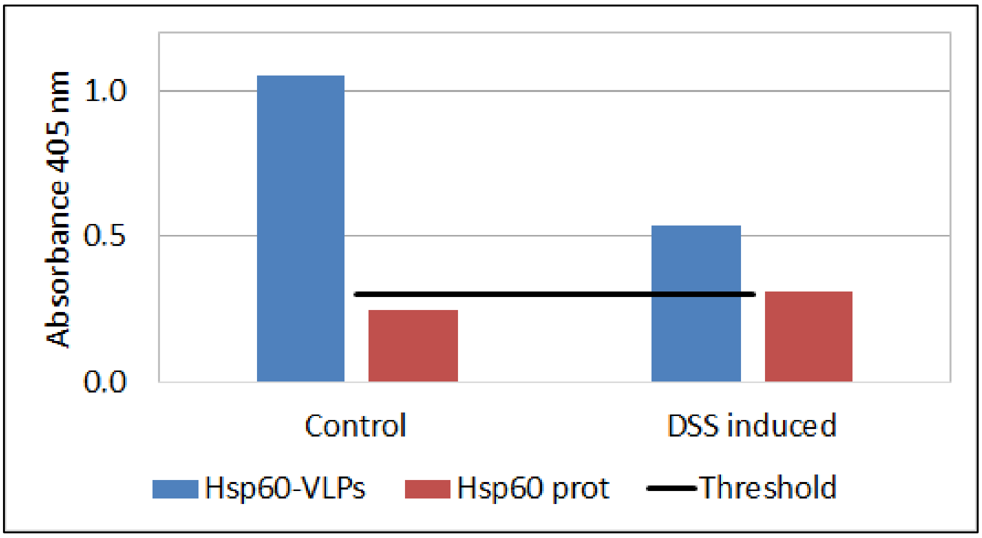

Figure S 5. Autoantibody levels in the sera measured with Hsp60-VLPs (blue) and Hsp60 protein (red). "Control" corresponds to healthy mice (n=5) and "DSS induced" corresponds to DSS-inflamed mice (n=5) mixed in a pool. Representative data of two detections are shown.
